# Involvement of community pharmacists in public health priorities: A multi-center descriptive survey in Ethiopia

**DOI:** 10.1371/journal.pone.0180943

**Published:** 2017-07-13

**Authors:** Daniel Asfaw Erku, Amanual Getnet Mersha

**Affiliations:** 1 Department of Clinical Pharmacy, School of Pharmacy, College of Medicine and Health Sciences, University of Gondar, Gondar, Ethiopia; 2 Department of Gynecology and Obstetrics, School of Medicine, College of Medicine and Health Sciences, University of Gondar, Gondar, Ethiopia; Universiti Sains Malaysia, XX

## Abstract

Located in the heart of the community and widely distributed geographically, community pharmacies provide a platform for a more proactive involvement in public health services. So far, little information has been gathered in Ethiopia on community pharmacists’ level of involvement in public health services. The aim of the present study was, therefore, to document the level of involvement of community pharmacy professionals in the provision of public health services and the barriers to such involvement. This study employed a self-administered questionnaire based survey, which asked participants to indicate their frequency and level of involvement in providing public health services and their perceived barriers in providing such services. Surveys were undertaken from May to July, 2016 with 472 community pharmacy professionals working in community pharmacies in six cities of Amhara regional state of Ethiopia: Debre Markos, Gondar, Dessie, Bahir Dar, Woldya and Debre Birhan. Among 472 community pharmacy professionals approached, 412 (233 pharmacists and 179 pharmacy technicians) completed the survey with a response rate of 87.3%. Most respondents reported as being either “not at all involved” or “little involved” in counselling on smoking cessation (79.3%), and screening for hypertension (86.9%), diabetes (89.5%), and dyslipidemia (88.9%). On the other hand, they reported a higher level of involvement in the management and screening of infectious diseases (72.8%) and counseling with partners when initiating treatment for sexually transmitted diseases (68.9%). Lack of knowledge or clinical skills and lack of personnel or resources were the most commonly reported barrier for expanding such services. This survey revealed a low level of involvement of community pharmacists in public health services. In order to better integrate community pharmacies into future public health programs and optimize the contribution of community pharmacy professionals, interventions should focus on overcoming the identified barriers.

## Introduction

One of the main agenda set by the World Health Organization (WHO) for the future of public health is to form accessible, multidisciplinary networks of public health professionals who actively engage within communities and provide key public health services in order to ultimately improve the life expectancy of the population [1]. Community pharmacy professionals, being one of the most easily accessible and widely distributed health professionals among the community, could be an indispensable component of this agenda. Community pharmacies have great potential as a setting in public health owing to their location in the heart of the community. This characteristic feature provides a platform for more proactive contribution in solving gaps in public health services and programs including health-promotion and a variety of preventive services [2]. Some of the health-promotion and preventive services which could be delivered via community pharmacy settings are promoting health and wellbeing (e.g. physical activity, weight management, curbing antibiotic resistance through promoting different community-based antimicrobial stewardship programs), preventing the occurrence of diseases (e.g. smoking cessation, infectious diseases), identifying and referring ill individuals via screening and maintaining health of those with chronic diseases such as diabetes, hypertension. The benefits of pharmacy services in these important public-health issues have been documented in many reviews and clinical trials [3–6].

However, there are a number of barriers that have hindered the provision of public health services in community pharmacy settings. Lack of knowledge and skills, lack of confidence and adequate training, lack of policies, poor recognition within the healthcare system, patients’ reluctance to use pharmacy services and presence of inadequate number of pharmacy staff are some of the factors which can contribute to the low level of pharmacy services uptake and public health initiatives [7–11]. In developed countries, such as the United Kingdom, pharmacists are well integrated into public-health programs [12]. In contrast, community pharmacies in Ethiopia are not yet legally included as a health care professionals within the public health workforce and their role in providing public health services is yet to be appropriately recognized and endorsed by public health and governmental agencies, academicians and other healthcare professionals. With much smaller numbers, compared to developed countries, community pharmacy professionals in developing countries including Ethiopia tend to focus on the traditional medication dispensing roles and seldom provides health prevention and promotion services. Taking the global evidence into consideration and due to lack of data in Ethiopia, the present study was conducted with the aim of documenting the level of involvement of pharmacists and pharmacy technicians in the provision of public health services and the perceived barriers that limit their involvement in the delivery of such services. Such data are essential to the improvement of public-health programs in community pharmacy in Ethiopia. Although there is a general understanding of the way CDROs are providing population-based services to the public as well as barriers that hinders to the provision of such services, there is limited quantitative data in Ethiopia on the specific public health priorities in which pharmacy professionals are involved. The finding of our study will, therefore, fill the existing literature gap in Ethiopia and inform policy makers, public health agencies and other healthcare professionals regarding community pharmacy based public health services and potential barriers so as to better integrate community pharmacy professionals in the public health taskforce.

## Materials and methods

### Study design and setting

In this cross-sectional survey, a self-administered questionnaire was distributed to all community pharmacists and pharmacy technicians practicing in community drug retail outlets (CDROs) of 6 selected cities of Amhara regional state, the second most populous region among the nine regional states of Ethiopia. As the roles and responsibilities of pharmacy technicians in pharmacy practice is significant in Ethiopia, we included pharmacy technicians in the survey. The region covers an area of 154,709 km^2^ and the region is divided into 11 zones for administrative purpose [13]. This survey was conducted in Debre Markos (referral center for west Gojjam), Gondar (referral center for North and South Gondar), Dessie (referral center for South Wollo and Oromia special zone), Bahir Dar (referral center for West Gojjam and Awi zones), Woldya (referral center for North Wollo) and Debre Birhan (referral center for North Shewa).

### Sample size

The CDROs in Ethiopia are divided into pharmacy, drug store and rural drug vendor based on the kind of medications they are supposed to dispense and the qualification of service providers. Pharmacies run only by a pharmacist (with qualification of a university degree or above), drug shop run by druggist (with qualification of diploma in pharmacy) and rural drug vendor run by health assistants. According to the recent report by Federal Ministry of Health (FMOH) Health Sector Development Program (HSDP) IV (2010–2015), there were a total of 661 active CDROs in the country at the end of 2010. However, there is no available data on the proportion of pharmacists and pharmacy technicians currently practicing in each of the cities and regions. Thus, all of the consenting community pharmacists and pharmacy technicians working in the aforementioned cities were included in this survey, while community pharmacy professionals working in acute- and chronic-care hospitals and those who refuse to participate were excluded. Community pharmacy professionals working in more than one CDROs were asked to refer to the one in which they worked most of the time. Accordingly, a total of 472 community pharmacy professionals (233 pharmacy technicians and 229 pharmacists) were invited to participate in the survey.

### The study tool

The questionnaire was created by modifying items in three previously used instruments regarding the role of community pharmacists in health education and disease prevention [14, 15 and 16], and items were thoroughly reviewed for relevance by a team of experts including experienced pharmacists and public health experts. The survey instrument was further validated by pilot-testing on 20 voluntary community pharmacists working in CDROs of the designated study areas. After collecting feedback from the participating community pharmacists, which were not included in the final survey, slight modification was instituted in the final data collection tool.

This survey collected data in two major areas. The first area surveyed was the public health services (health-promotion and preventive) that community pharmacies are providing which contribute to a better public health and level of involvement in such services (“very involved” to “not at all involved”). We classify these services into Lifestyle (which includes smoking cessation, physical-activity promotion, healthy eating, weight management and alcohol consumption), Screening (including hypertension, diabetes, dyslipidemia and risk of suicide) and Miscellaneous public health services which includes involvement in the management and screening of infectious diseases, promoting antimicrobial stewardship programs, counseling with partners when initiating treatment for sexually transmitted diseases, counseling on emergency and other type of contraception and conducting needs assessments to identify health risks in the community. Respondents were asked to indicate the frequency of the services provided as well as the staff involved in the delivery (pharmacist, pharmacy technician or others). Respondents’ perceived barriers in the delivery of such services in their practice settings were then asked in section two of the questionnaire which includes lack of knowledge or clinical skills, lack of personnel or resources, lack of clinical tools, lack of coordination with other health care professionals, lack of access to additional training programs, lack of time, patients are not interested in preventive activities and others (lack of financial compensation, lack of space and patients generally have more urgent medical conditions). The survey instrument, along with a cover letter, is provided in supporting information (S1 File).

### Statistical analyses

The data collected were entered into and analyzed using Statistical Package for the Social Sciences (SPSS) software version 21.0 for Windows (SPSS Inc., Chicago, IL). Shapiro-Wilk test was employed to assess the normality of the data. Means with standard deviations (SD) for continuous variables and proportions for discrete variables were utilized. For the section on community pharmacy professionals’ involvement in public health services and the perceived barriers to the provision of such services, the proportions of respondents selecting each possible answer were first computed. The association between their involvement and various demographic variables were compared by using Pearson’s chi-square test. Student t-test were also employed for computing differences among CDROs (drug store and pharmacy). A *p*-value of less than 0.05 was considered as statistically significant.

### Ethical clearance

This study was approved by the ethical committee of University of Gondar with a references number of UoG-SoP/0864/2016. Written informed consent from all participants were also gained before commencing the study. Participants’ information obtained was kept confidential.

### Result

Among 472 community pharmacy professionals approached, 412 (233 pharmacists and 179 pharmacy technicians) completed the survey with a response rate of 87.3%. Among the respondents, 284 (68.9) were males, with a mean (with SD) age of 28.7±8.4 years. The characteristics of respondents and CDROs are shown in Table 1.

**Table 1:** Characteristics of community pharmacy professionals and CDROs, Amhara, Ethiopia, N = 412.

Characteristics	Frequency (%)
Mean Age (years)	28.7±8.4 (SD)
**Gender**	
Male	284(68.9)
Female	128 (31.1)
**Highest pharmacy degree achieved**	
Diploma	179 (43.4)
Bachelors (B.Pharm)	200 (48.5)
Post-graduate	33 (8)
**Work experience in CDROs**	
< 5 years	251(60.9)
>5 years	161 (39.1)
**Practice region**	
Debre Markos	60 (14.6)
Gondar	71 (17.2)
Dessie	87 (21.1)
Bahir Dar	78 (18.9)
Woldya	61 (14.8)
Debre Birhan	55 (13.3)
**Type of CDRO**	
Pharmacy	221 (53.6)
Drug store	126 (30.6)
Others*	65 (15.8)
**Average prescriptions filled per day**	
< 250	168 (40.8%)
250–500	113 (27.4)
> 500	131 (31.8)

Abbreviation: CDRO; community drug retail outlet

*Others include pharmacy associated with supermarket and rural drug vendors

### Levels and characteristics of involvement in public health services

Levels of involvement and characteristics of public health services provided by community pharmacy professionals working in CDROs are tabulated in Tables 2 and 3 respectively. Most respondents reported their community pharmacy as being either “not at all involved” or “little involved” in counselling on smoking cessation (79.3%), weight management (69.6%), screening for hypertension (86.9%), diabetes (89.5%), dyslipidemia (88.9), risk of suicide (95.1%) and conduct needs assessments to identify health risks in the community (89.8%). The proportion of community pharmacy professionals who reported their community pharmacy as being “very involved” or “involved” in each service was 72.8% for involvement in the management and screening of infectious diseases, 68.9% for counseling with partners when initiating treatment for sexually transmitted diseases, 62.4% for promoting antimicrobial stewardship programs, 57.7% for counseling on emergency and other type of contraception, 53.9% for counselling on alcohol consumption and 53.6% for counselling on healthy eating.

**Table 2:** Levels of involvement of community pharmacy professionals in public health services, Amhara, Ethiopia, N = 412.

Services	Very involved, N (%)	Involved, N (%)	Little involved, N (%)	Not at all involved, N (%)
**Lifestyle**				
Counseling on smoking cessation	35 (8.5)	50 (12.1)	130 (31.5)	197 (47.8)
Physical-activity promotion	45 (10.9)	53 (12.9)	244 (59.2)	70 (17)
Healthy eating	123 (29.8)	98 (23.8)	70 (17)	121 (29.4)
Weight management	78 (18.9)	47 (11.4)	54 (13.1)	233 (56.5)
Alcohol consumption	140 (34)	82 (19.9)	78 (18.9)	112 (27.2)
**Screening for**:				
Hypertension	25 (6.1)	29 (7)	89 (21.6)	269 (65.3)
Diabetes	20 (4.8)	23 (5.6)	127 (30.8)	242 (58.7)
Dyslipidemia	13 (3.1)	33 (8)	79 (19.2)	287 (69.7)
Risk of suicide	5 (1.2)	15 (3.6)	57 (13.8)	335 (81.3)
**Miscellaneous public health services**				
Involved in the management and screening of infectious diseases	173 (42)	127 (30.8)	50 (12.1)	62 (15)
Promote antimicrobial stewardship programs	177 (43)	80 (19.4)	55 (13.3)	100 (24.3)
Counseling with partners when initiating treatment for sexually transmitted diseases (STDs)	218 (52.9)	66 (16)	46 (11.3)	82 (19.9)
Counseling on emergency and other type of contraception	144 (34.9)	94 (22.8)	79 (19.2)	95 (23)
Conduct needs assessments to identify health risks in the community	23(5.6)	19 (4.6)	53 (12.9)	317 (76.9)

**Table 3:** Characteristics of public health services provided by community pharmacy professionals, Ethiopia, N = 412.

Variables	Lifestyle	Screening	Miscellaneous
**Main person(s) providing public health services, n (%)**			
Pharmacist	239 (58)	292 (70.9)	300 (72.8)
Pharmacy technicians	115 (27.9)	70 (17)	71 (17.2)
Others*	58 (14.1)	50 (12.1)	41 (9.9)
**Frequency of services provided, n (%)**			
Few times per week	45 (10.9)	38 (9.3)	189 (45.9)
Few times per month	173 (42)	144 (34.9)	161 (39.1)
Few times per year	194 (47.1)	230 (55.8)	62 (15)
**Specific activities conducted in public health services n (%)**			
Counseling when dispensing medications	315 (76.4)	180 (43.7)	220 (53.4)
Referral to government hospitals	59 (14.2)	164 (39.8)	167 (40.5)
Others**	38 (9.2)	68 (16.5)	25 (6.1)

* Others include pharmacy interns and health assistants

**Others include “Distribution of written information”, and “Personalized follow-up or private consultation”

Pharmacists were the frequently cited professionals as the main provider of public health services (58% for lifestyle, 70.9% for screening and 72.8% for miscellaneous services) in their CDROs followed by pharmacy technicians (27.9% for lifestyle, 17 for screening and 17.2 for miscellaneous services). Miscellaneous public health services were reported for the most part to take place a “few times per week” or a “few times per month” (85%), while screening and lifestyle were reported to take place a few times per year (55.8% and 47.1% respectively). Moreover, providing counseling when dispensing medications (76.4% for lifestyle, 43.7% for screening and 53.4% for miscellaneous services) and referring patients to referral government hospitals (14.2% for lifestyle, 39.8% for screening and 40.5% for miscellaneous services) were reported when asked what specific activities are conducted while delivering public health services (Table 3). Analysis were done on the association between involvement of community pharmacy professionals in public health services and different demographic variables. Accordingly, community pharmacy professionals with a qualification of degree or above had a significantly higher level of service delivery than those having diploma in pharmacy with regard to sexual health (*p* value 0.001), infectious disease (*p* value<0.020), and promotion of healthy lifestyle (*p* value<0.04). Similarly, community pharmacy professionals with more than 5 years of experience had a higher involvement in screening for diabetes (*p* value<0.030) and hypertension (*p* value<0.000), infectious disease (*p* value<0.031) and sexual health (*p* value<0.014) compared to those with less than 5 years of experience. No statistically significant associations were found between involvement in public health services and other demographic variables such as age and gender (Table 4).

**Table 4:** Statistical test (chi square) of the involvement of community pharmacy professionals in selected public health priorities according to demographic variables, Amhara, Ethiopia (N = 412).

Services *	Lifestyle	Screening for hypertension	Screening for diabetes	Screening for dyslipidemia	Sexual health	Infectious disease
**Gender**						
Male	136 (33%)	85 (20.6%)	109 (26.4%)	76 (18.4%)	210 (51%)	221 (53.6%)
Female	79 (19.2%)	58 (14.1%)	61 (14.8%)	49 (11.9%)	102 (24.7%)	109 (26.4%)
*p*-value	0.581	0.210	0.310	0.061	0.081	0.530
**Work experience**						
<5 years	83 (20.1%)	42 (10.2%)	78 (18.8%)	36 (8.7%)	164 (39.8%)	189 (45.9%)
>5 years	132 (32%)	101 (24.5%)	92 (22.4%)	89 (21.6%)	148 (35.9%)	141(34.2%)
*p*-value	0.571	0.000**	0.030**	0.060	0.014**	0.031**
**Educational level**						
Diploma	45 (10.9%)	51 (12.4%)	76 (18.4%)	20 (4.8%)	120 (29.1%)	149 (36.2%)
Degree and above	170 (41.3%)	92 (22.3%)	94 (22.8%)	105 (24.5%)	192 (46.6%)	181 (43.9%)
*p*-value	0.040**	0.061	0.091	0.073	0.001**	0.020**

* frequency reported are the proportion of community pharmacy professionals who provide (regardless of the extent) public health services;

**statistically significant association (*p*-value<0.05)

### Perceived barriers to the provision of public health services

As depicted in Table 5, lack of knowledge or clinical skills was the most commonly reported barrier for delivering public health services in the pharmacy followed by lack of access to additional training programs, lack of personnel or resources, and lack of coordination with other health care professionals. Four items, lack of personnel or resources, lack of access to additional training programs, lack of knowledge or clinical skills and lack of clinical tools were more commonly perceived as serious problems in drug stores than in pharmacies (*p* value<0.05).

**Table 5:** Perceived barriers to the provision of public health services in CDROs (Mean) (Likert scale: 0 = not at all problematic to 5 = extremely problematic), Amhara, Ethiopia, N = 412.

Perceived barriers	Type of CDROs		Total	*P* value
	Drug store	Pharmacy		
Lack of knowledge or clinical skills	4.32	4.03	4.17	<0.001**
Lack of personnel or resources	4.11	3.54	3.82	0.412
Lack of clinical tools	3.63	3.12	3.37	0.026**
Lack of coordination with other health care professionals	3.54	3.97	3.75	0.043 **
Lack of access to additional training programs	4.07	3.89	3.98	<0.001**
Lack of time	3.91	3.06	3.48	0.234
Patients are not interested in preventive activities	3.29	2.65	2.97	0.129
Others^a^	3.66	3.14	3.4	0.153

^a^Others include lack of financial compensation, lack of space and patients generally have more urgent medical conditions;

**statistically significant association (p-value<0.05)

## Discussion

The changing role of community pharmacy professionals from their traditional dispensing responsibilities to a greater contribution to population health is being accepted all over the globe [17]. To the best of our knowledge, this is the first survey to explore the extent and type of involvement of community pharmacy professionals in public health priorities in Ethiopia.

According to the finding of this survey, community pharmacy professionals are less intensely engaged in the provision of public health services. Although the beneficial effect of involving community pharmacy professionals in public-health activities such as smoking cessation, hypertension, diabetes, and sexual health has been confirmed in different clinical trials [17, 18], it’s apparent from our study that community pharmacy professionals’ involvement in Ethiopia is severely limited. The findings of this survey were supported by similar studies done elsewhere which showed that community pharmacy professionals are mostly involved in activities pertaining to the dispensing of medications and have less intense involvement in public health activities [19, 20]. Community pharmacy professionals in this study were less intensely engaged in the area of smoking cessation, weight management and screening for hypertension, diabetes, dyslipidemia and risk of suicide. In a study done in Northern Ireland to evaluate the role of a community pharmacies in smoking cessation, the involvement of community pharmacy professionals was especially low (19%) [21]. This is consistent with the findings of our result, where only 8.5% of respondents reported provision of counselling on smoking cessation. However, the involvement of community pharmacy professionals in chronic disease screening and lifestyle counselling were found to be very low compared to studies done in developed countries such as Canada and USA, where community pharmacy professionals provide a variety of diagnostic screening services for blood pressure, dyslipidemia, and capillary glucose as well as pharmaceutical and preventive care services for asthma, diabetes, and hypertension [16–19]. This is due to the narrow scope of Ethiopian community pharmacy professionals as they are largely limited to the dispensing of medications and seldom provides screening and counseling services on chronic non-communicable diseases. While some community pharmacies are offering pharmaceutical care to chronically ill patients, the role is still in its infant stage. Their responsibilities in chronic disease state management and health promotion should be further developed to successfully tap their invaluable contribution to patient care. On the other hand, community pharmacy professionals in this survey reported a higher level of involvement in sexual health and management and screening of infectious diseases. Similar studies conducted elsewhere also reported a higher involvement of community pharmacy professionals in sexual health including emergency contraception, treatment for vaginal candida infection, information on hepatitis B and HIV and sexual health promotion [22–24]. The results of this study corroborate with a national survey conducted in Ethiopia which reported a higher involvement of community pharmacies in promoting antimicrobial stewardship and infection prevention [25]. Given the high prevalence of infectious diseases and availability of continuous trainings on tropical and communicable diseases, it is not surprising that pharmacy professionals in Ethiopia are well engaged in the provision of public health services pertaining to infectious and communicable diseases.

Furthermore, it has been found that community pharmacy professionals with a qualification of degree or above and with more than 5 years of experience had a significantly higher level of public health service delivery than those having diploma and with less than 5 years of experience. With the recent adoption of ward-based clinical pharmacy training in undergraduate pharmacy schools of Ethiopia, along with a higher number of years working in community pharmacy settings, it’s not surprising that pharmacy professionals having a bachelor and/or postgraduate degree and more than 5 years of experience in practice settings had a relatively higher level of involvement in public health services.

Integrating CDROs into regional and national population health programs is a valuable and crucial way for improving public health in the country. In line with this, understanding the various barriers for the provision of such services is the first key step in this community pharmacy-public health integration process. In the present survey, lack of knowledge or clinical skills was the most commonly reported barrier to the provision of public health service in community pharmacy followed by lack of access to additional training programs and lack of personnel or resources. Similar barriers have also been documented in different parts of the globe including lack of time and personnel, lack of clinical skills and tools [7–10]. Public’s lack of awareness of community pharmacy professional’s role in public health and uncooperative patients were other important barriers identified in the literature but had not been prominent in our study [26, 27]. Studies conducted in both developed and developing countries also reported that training programs are needed so as to boost community pharmacy professional’s confidence in providing public health services, making educational intervention a necessity [17, 28]. Providing specific trainings that will fill the knowledge and skill gap required for the provision of such services is recommended both in academia and practice settings as it will result in the delivery of public health services in a better and more skillful way than ever before. Overcoming the aforementioned perceived barriers also needs restructuring the health care system of the country so as to integrate these indispensable health care professionals into the public health task force. This restructuring entails beyond merely financial investments in the sector; full commitment and engagement also needs to be achieved by all pharmacy stakeholders, academic institutions, health policy makers and non-governmental organizations involved in the provision of public health. Moreover, considering compensation for service delivery, interdisciplinary communication, and re-statement of the role and responsibility of community pharmacy professionals may result in a better provision of community pharmacy based public health services.

## Strength and limitation of the study

This survey highlights an area of community pharmacy practice where there is lack of literature in Ethiopia. Yet, the survey has some limitations that should be taken into account while interpreting the results. As the study was a descriptive cross-sectional survey conducted in only six selected cities of Amhara regional state, caution should be exercised when generalizing to other cities and regions in Ethiopia. Moreover, our use of a self-administered questionnaire, which depends on honesty and faith of the respondents, could affect the responses as it may be subjected to respondent or recall bias. Even with the above limitations, this survey has significant implications for improving the involvement of community pharmacies in health promotion and prevention by serving as a general comparison of frequency and characteristics of delivery of public health services in different sites of the country.

## Conclusions

The present survey demonstrated that community pharmacy professionals’ involvement in public health services is low particularly in lifestyle-related counselling activities (smoking cessation, promoting physical-activity and healthy eating, weight management and alcohol consumption) and screening services (for hypertension, diabetes, dyslipidemia and risk of suicide). Lack of knowledge or clinical skills was the most commonly reported barrier for expanding public health service in community pharmacy settings. In order to better integrate community pharmacies into future public health programs and optimize the contribution of pharmacists, interventions should focus on overcoming the identified barriers. Follow-up studies seeking community pharmacy professionals’ involvement in public health services on a national scale may also be needed to identify more barriers and to better inform regulatory bodies.

## Supporting information

S1 File Data collection tool along with cover letter. (DOCX)

## Abbreviations

CDROs: community drug retail outlets
SD: standard deviation
SPSS: Statistical Package for the Social Sciences
WHO: World Health Organization
