# Supplementary material for: Involvement of community pharmacists in public health priorities: A multi-center descriptive survey in Ethiopia
Source: PLoS One. 2017 Jul 13;12(7):e0180943. doi: 10.1371/journal.pone.0180943 (PMC5509284; doi:10.1371/journal.pone.0180943)
Supplement: S1 File — (DOCX) [file pone.0180943.s001.docx]

**Cover letter**

**Consent Information Sheet**

My name is __________________________. I am here on behalf of School of Pharmacy research group staffs. We are conducting a research on **‘Involvement of community pharmacists in public health priorities: a multi-center descriptive survey in Ethiopia’** The research project is approved by the Research Ethics committee of School of Pharmacy, University of Gondar. Your participation is purely based on your willingness and you have the right to choose not to take part in this study. If you choose to take part, you have the right to stop at any time.

If you agree to participate in the study, you will be asked to answer some questions about yourself and your level of involvement in selected public health services as well as perceived barriers in the provision of such services. The interview with you will take about 20 minutes. The information that you provide will be kept confidential by using only code numbers and locking the data.

Based on the understanding of the information I gave you, are you willing to participate in this study?

1. Yes
2. No

**Part 1: Demographic characteristics**

| **Sr. No** | **Questions** | **Choice Answers** |
| --- | --- | --- |
| 101 | Gender | 1. Male 2. Female |
| 102 | Age | in years: __________ |
| 103 | Experience | in years: __________ |
| 104 | Practice region | 1. Debre Markos 2. Gondar 3. Dessie 4. Bahir Dar 5. Woldya 6. Debre Birhan |
| 105 | Average prescriptions filled per day | _______________ |
| 106 | Type of CDRO | 1. Pharmacy 2. Drug store 3. Rural drug vendor 4. Chain Pharmacy |

**Part 2:** Indicate your levels of involvement of in the following selected public health services

| **Services** | **Very involved** | **Involved** | **Little involved** | **Not at all involved** |
| --- | --- | --- | --- | --- |
| **Lifestyle** |  |  |  |  |
| Counseling on smoking cessation |  |  |  |  |
| Physical-activity promotion |  |  |  |  |
| Healthy eating |  |  |  |  |
| Weight management |  |  |  |  |
| Alcohol consumption |  |  |  |  |
| **Screening for:** |  |  |  |  |
| Hypertension |  |  |  |  |
| Diabetes |  |  |  |  |
| Dyslipidemia |  |  |  |  |
| Risk of suicide |  |  |  |  |
| **Miscellaneous public health services** |  |  |  |  |
| Involved in the management and screening of infectious diseases |  |  |  |  |
| Promote antimicrobial stewardship programs |  |  |  |  |
| Counseling with partners when initiating treatment for sexually transmitted diseases (STDs) |  |  |  |  |
| Counseling on emergency and other type of contraception |  |  |  |  |
| Conduct needs assessments to identify health risks in the community |  |  |  |  |

**Part 3:** Characteristics of public health services provided (select from the choice given below)

| **How often do you provide those services** | 1. Few times per week 2. Few times per month 3. Few times per year |
| --- | --- |
| **Who provides the services?** | 1. Pharmacists 2. Pharmacy technicians 3. Pharmacy interns 4. Health assistants 5. Others, please specify_________ |
| **Which of the following specific activities do you provide as part of the public health services** | 1. Counseling when dispensing medications 2. Referral to government hospitals 3. Distribution of written information 4. Personalized follow-up or private consultation 5. Others, please specify__________ |

**Part 4:** Please rate the following perceived barriers that may hinder you from proving such services (0= not at all problematic to 5= extremely problematic)

| **Perceived barriers** | **1** | **2** | **3** | **4** | **5** |
| --- | --- | --- | --- | --- | --- |
| Lack of knowledge or clinical skills |  |  |  |  |  |
| Lack of personnel or resources |  |  |  |  |  |
| Lack of clinical tools |  |  |  |  |  |
| Lack of coordination with other health care professionals |  |  |  |  |  |
| Lack of access to additional training programs |  |  |  |  |  |
| Lack of time |  |  |  |  |  |
| Patients are not interested in preventive activities |  |  |  |  |  |
| Lack of financial compensation |  |  |  |  |  |
| Lack of space |  |  |  |  |  |
| Patients generally have more urgent medical conditions |  |  |  |  |  |

**Thank you for your participation!!!**
